# Supplementary material for: Evaluation of a Medical Grade Thermoplastic Polyurethane for the Manufacture of an Implantable Medical Device: The Impact of FDM 3D-Printing and Gamma Sterilization
Source: Pharmaceutics. 2023 Jan 30;15(2):456. doi: 10.3390/pharmaceutics15020456 (PMC9960613; doi:10.3390/pharmaceutics15020456)
Supplement: Supplementary file 1 [file pharmaceutics-15-00456-s001.zip › pharmaceutics-2114308-supplementary.pdf]

pharmaceutics

MDPI

Supplementary Materials:

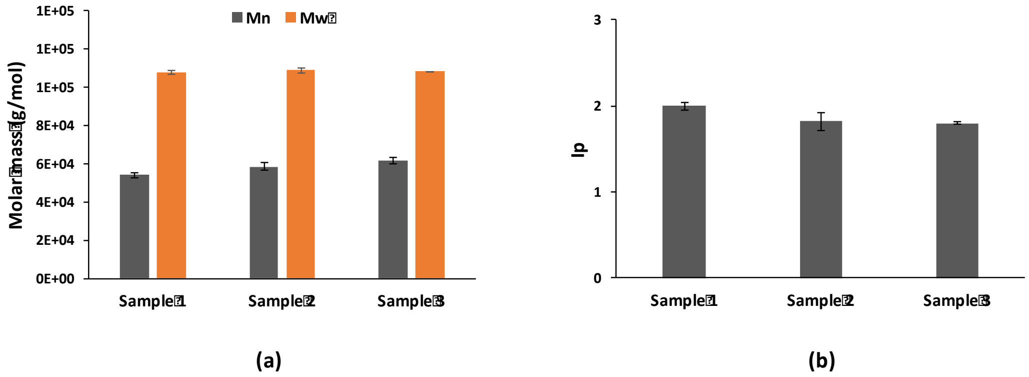

Figure S1. Reproducibility of FDM processing: Molar mass (a) and Polydispersity Index Ip (b) of samples after one, two and three successive prints (n = 2).
